# Investigating cannabinoids as P2X purinoreceptor 4 ligands by using surface plasmon resonance and computational docking

**DOI:** 10.1016/j.heliyon.2023.e21265

**Published:** 2023-10-21

**Authors:** Tess Puopolo, Ang Cai, Chang Liu, Hang Ma, Navindra P. Seeram

**Affiliations:** Department of Biomedical and Pharmaceutical Sciences, College of Pharmacy, University of Rhode Island, Kingston, RI 02881, USA

## Abstract

P2X purinoceptor 4 (P2X4) is an ATP-gated ion channel receptor with diverse neurophysiological functions, and P2X4 modulators hold promise as potential therapeutics for neuropathic pain, neuroinflammation, and neurodegenerative diseases. While several cannabinoids have been reported as modulators of purinoreceptors, their specific purinoreceptor-binding characteristics remain elusive. In this study, we established a comprehensive workflow that included a binding screening platform and a novel surface plasmon resonance (SPR) competitive assay, complemented by computational docking, to identify potential P2X4 binders among a panel of twenty-eight cannabinoids. Through SPR, we determined the binding affinities of cannabinoids (*K*_D_ values ranging from 3.4 × 10^−4^ M to 1 × 10^−6^ M), along with two known P2X4 antagonists, BX430 (*K*_D_ = 4.5 × 10^−6^ M) and 5-BDBD (*K*_D_ = 7.8 × 10^−6^ M). The competitive SPR assay validated that BX430 and 5-BDBD acted as non-competitive binders with P2X4. In the following competitive assays, two cannabinoids including cannabidiol (CBD) and cannabivarin (CBV) were identified as competitive P2X4-binders with 5-BDBD, while the remaining cannabinoids exhibited non-competitive binding with either BX430 or 5-BDBD. Our molecular docking experiments further supported these findings, demonstrating that both CBD and CBV shared identical binding sites with residues in the 5-BDBD binding pocket on P2X4. In conclusion, this study provides valuable insights into the P2X4-binding affinity of cannabinoids through SPR and sheds light on the interactions between cannabinoids (CBD and CBV) and P2X4.

## Graphical abstract

**Figure:**
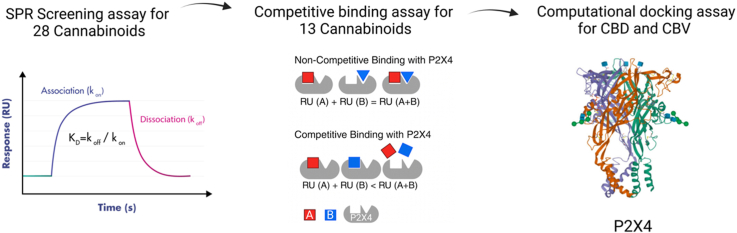

## 1 Introduction

The P2X purinoreceptor 4 (P2X4) is predominantly expressed in the central nervous system and immune system cells, implicating its crucial role in neuroinflammation [1]. Upon activation, P2X4 triggers a calcium ion influx, leading to depolarization and downstream signaling cascades [2,3]. Notably, P2X4 is one of seven non-selective cation channel receptors in the P2X family and has been found to form heterotrimers with other P2X receptors, such as P2X7 [2]. P2X4's specific location and increased expression upon injury has brought it to the forefront as a promising target of interest [4]. Studies have linked P2X4 to the modulation of essential neurotransmitter systems, including gamma-aminobutyric acid and glutamate, as well as microglial activation in neuropathic pain and its upregulation in neurodegenerative diseases [3,[5], [6], [7]]. These multifactorial effects potentially contribute to its neuroprotective effects, functioning through a neuroimmune mechanism [4]. Given its significance in various neuro-related pathological conditions, exploring novel small molecule P2X4 ligands may hold promising therapeutic value [3].

Despite the discovery of small molecule antagonists targeting P2X4, their development as viable drug candidates have encountered obstacles due to challenges such as poor solubility, species variance, and potency [1,8]. For instance, 5-BDBD, characterized as a selective competitive P2X4 antagonist in HEK293 cells expressing human P2X4 (IC_50_ = 1.2 μM) [9], has also been found to exhibit a non-competitive binding manner in radioligand binding assays, indicating discrepancy between studies [2]. Similarly, TNP-ATP, another P2X4 antagonist, shows competitive binding affinity, but lacks strong selectivity towards P2X4 over other P2X subtypes [2]. Additionally, BX430, identified as a selective non-competitive (allosteric) antagonist of P2X4, exerts a unique action by inhibiting P2X4 pore formation, yet exhibits differences in efficacy amongst species limiting its translational potential (IC_50_ = 0.54 μM) [2].

Given the absence of clinically approved P2X4 inhibitors, the exploration of novel compounds from natural resource is crucial. Phytochemicals derived from medicinal plants, in particular, hold promise as potential P2X4 modulators, given their effects on the P2X family, including P2X4 [[10], [11], [12], [13], [14], [15]]. Notably, cannabinoids from Cannabis (*Cannabis sativa* L.) have been reported for the ameliorative effects against neuroinflammation, neuropathic pain, and nervous system disorders [16]. Previous studies have shown that cannabinoids’ effects on psychosis risks are associated with their modulations of a P2X purinoceptor (P2X7) [17]. Additionally, our group has previously reported the inhibitory effect of the non-psychoactive phytocannabinoid, namely, cannabidiol (CBD), on the inflammasome activation via the modulation of P2X7 [18]. However, it remains unclear whether CBD and other cannabinoids can also modulate additional P2X receptors, such as P2X4. Therefore, this further investigation into the potential modulatory effects of CBD and other cannabinoids on P2X4 could pave the way for novel therapeutic interventions targeting P2X4-related pathologies.

In this study, our objective is to establish a binding screening platform utilizing the biophysical technique of surface plasmon resonance (SPR). The primary goal is to investigate the binding affinity between a panel of cannabinoids, in addition to two known antagonists, BX430 and 5-BDBD, with the P2X4 receptor. To gain a comprehensive understanding of the drug binding pockets, we have developed a novel SPR competitive assay. This assay allows us to analyze the binding patterns of cannabinoids and known antagonists. Through this approach, we can categorize cannabinoids as competitive or non-competitive binders, based on their binding profile with BX430 and 5-BDBD, respectively. Moreover, we aim to uncover insights into the binding mechanism by probing the potential binding sites of the competitive cannabinoids in comparison to the antagonist, 5-BDBD. This research seeks to contribute valuable knowledge to the field of cannabinoid interactions with the P2X4 receptor and their potential implication.

## 2 Material and methods

### 2.1 Chemicals and reagents

Purinergic receptor P2X, ligand gated ion channel 4 (P2RX4) protein, the extracellular domain construct (77–338 amino acid sequence) with an N-terminal His tag was purchased from MyBioSource, Inc (San Diego, CA, USA). Series S SPR sensor chip CM5 was obtained from Cytiva Life Sciences (Chicago, IL, USA). Polypropylene Greiner Bio-One microplates (384-well) were purchased from VWR, Avantor Sciences (Radnor, PA, USA). Amine coupling kit, HBS-EP+ (0.1 M HEPES, 1.5 M NaCl, 0.03 M EDTA and 0.5 % v/v surfactant P20), polypropylene vials, rubber caps, and 384 well microplate foils were purchased from Cytiva Life Sciences (Chicago, IL, USA). Phosphate buffered saline (PBS) and dimethyl sulfoxide (DMSO) were obtained from Thermo Fisher Scientific (Waltham, MA, USA).

**Fig. 1:**
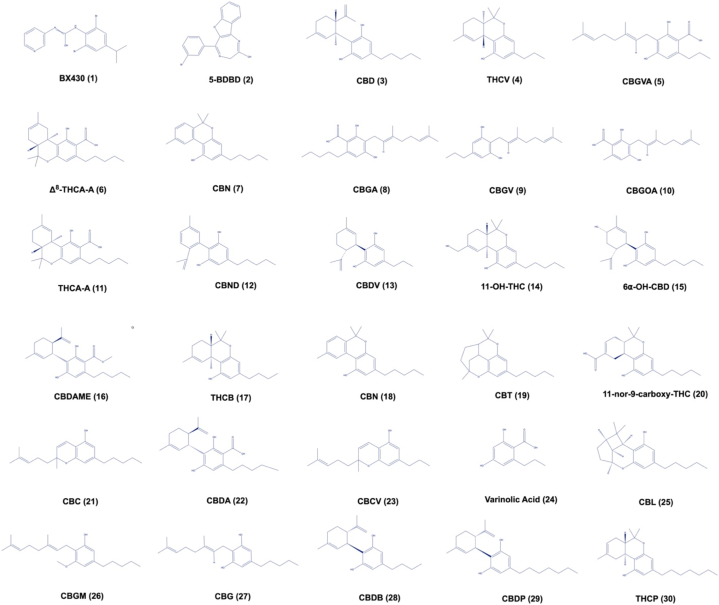
Chemical structures of controls (BX430 (1) and 5-BDBD (2)) and evaluated cannabinoids CBD (3), THCV (4), CBGVA (5), Δ^8^-THCA-A (6), CBV (7), CBGA (8), CBGV (9), CBGOA (10), THCA-A (11), CBND (12), CBDV (13), 11-OH-THC (14), 6α–OH–CBD (15), CBDAME (16), THCB (17), CBN (18), CBT (19), 11-nor-9-carboxy-THC (20), CBC (21), CBDA (22), CBCV (23), varinolic acid (24), CBL (25), CBGM (26), CBG (27), CBDB (28), CBDP (29), and THCP (30).

The positive controls (BX430, and 5-BDBD) were purchased from Cayman Chemical Company (Ann Arbor, MI, USA). Cannabinoids were purchased from Cayman Chemical Company (Ann Arbor, MI, USA), including CBD (cannabidiol), THCV (Δ^9^-tetrahydrocannabivarinic acid A), CBGVA (cannabigerovarinic acid), Δ^8^-THCA-A (Δ^8^-trans-tetrahydrocannabinolic acid A), CBV (cannabivarin), CBGA (cannabigerolic acid), CBGV (cannabigerovarin), CBGOA (cannabigerorcinic acid), THCA-A (Δ^9^-tetrahydrocannabinolic acid A), CBND (cannabinodiol), CBDV (cannabidivarin), 11-OH-THC (11-nor-Δ^8^-tetrahydrocannabinol-9-carboxylic acid), 6α–OH–CBD (6α-hydroxy cannabidiol), CBDAME (cannabidiolic acid methyl ester), THCB (Δ^9^-tetrahydrocannabibutol), CBN (cannabinol), CBT (cannabicitran), 11-nor-9-carboxy-THC (11-nor-9-carboxy-Δ^8^-tetrahydrocannabinol), CBC (cannabichromene), CBDA (cannabidiolic acid), CBCV (cannabichromevarin), varinolic acid, CBL (cannabicyclol), CBGM (cannabigerol monomethyl ether), CBG (cannabigerol), CBDB (cannabidibutol), CBDP (cannabidiphorol), and THCP (Δ^9^-tetrahydrocannabiphorol). Fig. 1 shows the chemical structures of the positive controls (BX430 (1) and 5-BDBD (2)) and cannabinoids, CBD (3), THCV (4), CBGVA (5), Δ^8^-THCA-A (6), CBV (7), CBGA (8), CBGV (9), CBGOA (10), THCA-A (11), CBND (12), CBDV (13), 11-OH-THC (14), 6α–OH–CBD (15), CBDAME (16), THCB (17), CBN (18), CBT (19), 11-nor-9-carboxy-THC (20), CBC (21), CBDA (22), CBCV (23), varinolic acid (24), CBL (25), CBGM (26), CBG (27), CBDB (28), CBDP (29), and THCP (30).

### 2.2 P2X4 immobilization

Recombinant human P2X4 (500 μg/mL stock solution in Dnase free water) was diluted to 40 μg/mL in sodium acetate 4.0. The protein was immobilized on a CM5 chip based on an amine coupling method as previously described with minor modification [19]. Briefly, the immobilization method involved five phases. First, a stable baseline was established, and a response was observed. Next, ethanolamine hydrochloride (1 M; pH 8.5) was performed, and the P2X4 protein response was observed with a contact time of 2100s and flow rate of 10 μL/min. Both binding association and dissociation were identified as ethanolamine hydrochloride (1 M; pH 8.5) deactivated unreacted NHS-esters and removed electrostatically bound ligands with a contact time of 420 s and a flow rate of 10 μL/min. Completion of all five phases indicated successful immobilization of the P2X4 protein on the SPR sensor chip.

### 2.3 Surface plasmon resonance binding screening assay

**Fig. 2:**
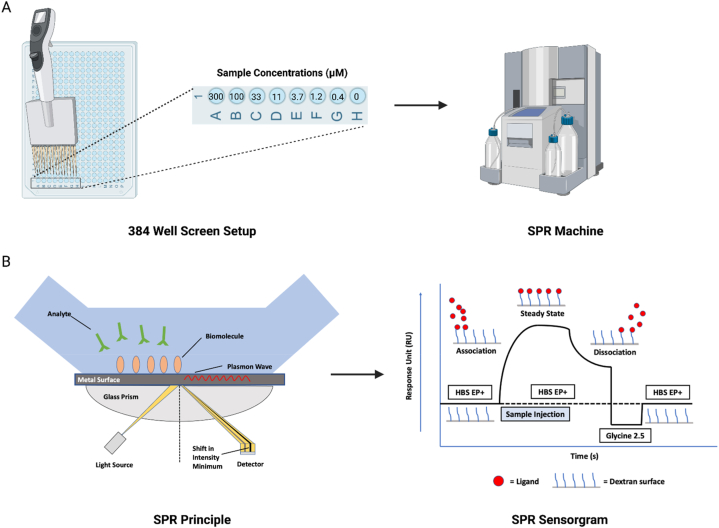
**Workflow and principle of the 384-well plate SPR screening assay with sensorgram schematic.** A) The 384-well plate is prepared with 3-fold sample dilutions in for input into SPR machine. Cannabinoid samples screened in this assay encompassed CBD, THCV, CBGVA, Δ^8^-THCA-A, CBV, CBGA, CBGV, CBGOA, THCA-A, CBND, CBDV, 11-OH-THC, 6α–OH–CBD, CBDAME, THCB, CBN, CBT, 11-nor-9-carboxy-THC, CBC, CBDA, CBCV, Varinolic acid, CBL, CBGM, CBG, CBDB, CBDP, and THCP. B) The principle of SPR methodology and a schematic representation of an SPR sensorgram were illustrated.

An SPR assay was conducted in a 384 well plate on the Biacore T200 SPR instrument (Cytiva; Marlborough, MA, USA) to screen a panel of cannabinoids (structures in Fig. 1) for P2X4 binding (Fig. 2A and B). PBS (20 mM) containing 3 % DMSO was used as the running buffer. Two P2X4 antagonists, BX430 and 5-BDBD, as well as the panel of cannabinoids were prepared at 10 mM in DMSO and diluted into a series of concentrations from 0.4 to 300 μM in the running buffer. The selection of this broad concentration range is attributed to the unknown binding affinity of cannabinoids. This range increases the likelihood of encompassing both 10-fold and 1/10-fold of the *K*_D_ value. Each sample was individually analyzed to assess its binding affinity to the P2X4 receptor. The regeneration solution (Glycine 2.5) was used after each sample cycle to eliminate any bound compounds, followed by a carry-over control (PBS containing 3 % DMSO) to prevent buffer mismatch in the subsequent sample cycle. To compensate for the bulk effects of DMSO, solvent correction cycles consisting of eight correction points (2.5–3.8 % of DMSO) were conducted before and after every thirty sample cycles. BIA Evaluation Software Version 4.1 (GE Healthcare, Chicago, IL, USA) was used to obtain binding parameters through a kinetics/affinity surface bound analysis. The CM5 chip's blank cell (flow cell 1) was subtracted from flow cell 2, which was immobilized with P2X4 protein, for each compound. Furthermore, the sensorgrams originating from the zero concentrations were subtracted to compensate for any systematic disturbances. A 1:1 binding mode was chosen as the default for analysis. Rigorous quality control measures were employed, and the BIA Evaluation Software was utilized to determine kinetic constants both within measurable limits and uniquely determined, thereby ensuring the reliability of the results. Binding parameters obtained included the association rate (k_on_), dissociation rate (k_off_) and the equilibrium dissociation constants (*K*_D_). All raw data (.txt) were exported from BIA Evaluation Software Version 4.1 and then imported into GraphPad Prism for figure generation.

### 2.4 Surface plasmon resonance competitive binding assay

A competitive SPR binding assay was designed to narrow down the cannabinoids’ binding site on P2X4. Theoretically, if the response units (RU) of molecule A (RU(A)) and molecule B (RU(B)) are individually lower than the combined response units when both A and B are present (RU (A + B)), it suggests a competitive binding scenario between A and B for the P2X4 receptor. Conversely, if the sum of RU(A) and RU(B) is equal to the response units when both A and B are present (RU (A + B)), a non-competitive binding model is suggested for A and B on the P2X4 receptor. First of all, we validated this competitive binding assay by comparing two known P2X4 antagonists, BX430 and 5-BDBD, using our previously reported method with modifications [19,20]. In addition, the conflicting reports on the binding mode of 5-BDBD, either as a competitive ATP active site binder [21], or an allosteric binder on P2X4 [22], prompted us to conduct a competitive SPR assay between 5-BDBD and BX430. Both compounds were dissolved in DMSO at 10 mM and diluted to 10 μM in PBS (20 mM). Both compounds, referred to as antagonists, were initially dissolved in DMSO at a concentration of 10 mM. Subsequently, they were diluted to a working concentration of 10 μM using PBS (20 mM) as the diluent. For individual runs, each antagonist was further diluted with the running buffer, composed of PBS (20 mM) with 3 % DMSO, resulting in a final concentration of 1 μM. To evaluate the interaction of both antagonists simultaneously, a 1:1 combination ratio was employed. Glycine, at a pH of 2.5 was utilized as the regeneration buffer. Additionally, solvent correction cycles were incorporated, consisting of eight correction points ranging from 2.5 % to 3.8 % of DMSO content, ensuring accurate data interpretation during the experimental procedure.

Subsequently, cannabinoids that demonstrated binding affinity to P2X4 during the initial screening were chosen for further investigation in a competitive assay, performed alongside both antagonists. Compounds were initially prepared at a concentration of 10 mM in DMSO and subsequently diluted in PBS (20 mM) to either 10 or 2 μM, depending on the binding response unit (RU) observed during the binding screening assays. For individual evaluations, compounds were diluted in a 1:1 combination ratio with the running buffer, PBS (20 mM) + 3 % DMSO, to either 5 or 1 μM.

To further explore the binding mode between cannabinoid with P2X4 antagonist (BX430 and 5-BDBD), a mixture of each cannabinoid (at 10 or 2 μM) and either BX430 or 5-BDBD (at 10 or 2 μM) was prepared in PBS (20 mM). These mixtures were subsequently diluted to either 5 or 1 μM using the running buffer. The experimental *K*_D_ values from the initial screening were used to calculate the fractional occupancy of each respective ligand using the following equation (Equation (1)):

(1) $$\text{FO}\;\left(A\right)=\frac{1}{1+\frac{K_{\mathrm{DA}}}{C_{A}}\;\left(1+\frac{C_{B}}{K_{\mathrm{DB}}}\right)};\;\text{FO}\;\left(B\right)\;\frac{1}{1+\frac{K_{\mathrm{DB}}}{C_{B}}\;\left(1+\frac{C_{A}}{K_{\mathrm{DA}}}\right)}$$

where C_A_ and C_B_ are the concentration of each binder and *K*_DA_ and *K*_DB_ are the equilibrium dissociation constants of each binder.

Further, the competitive theoretical RU of the respective cannabinoid with BX430 or 5-BDBD was computed utilizing the FO and R_max_ values using the following equation (Equation (2)):

(2) $$R_{\text{observed}}=\text{FO}\;\left(A\right)\cdot R_{\max}\;\left(A\right)+\text{FO}\;\left(B\right)\cdot {\text{R}}_{\text{max}}\;\left(B\right)$$

The theoretical non-competitive value was calculated as the sum of the experimental RU values of the cannabinoid and BX430, or the cannabinoid and 5-BDBD. The combined experimental RU value was compared to both the competitive and non-competitive theoretical RU values to determine the type of binding.

### 2.5 Computational docking

Based on the results from competitive binding assay, an in silico molecular docking was performed to explore the binding sites of the competitive cannabinoids CBD and CBV as analogous with P2X4's orthosteric site. To overcome the absence of a human P2X4 protein structure, the open state zebrafish P2X4 protein file (PDB ID: 4DW1) was obtained in pdb format from the RCSB Protein Data Bank (https://www.rcsb.org/). The ligand files for 5-BDBD, CBD and CBV were downloaded from the PubChem Database (https://pubchem.ncbi.nlm.nih.gov/) and converted into pdb files using UCSF Chimera. All files were dock prepped via the removal of water, addition of hydrogens, addition of charges, and assignment of AD4 type atoms. A grid box was created according to the orthosteric ATP binding site on P2X4 corresponding to the residues K70, K72, T189, and K193 on chain B, and N296, R298, and K316 on chain C [23]. All three ligands were independently docked to the P2X4 protein using the long genetic algorithm parameter with the maximum number of evaluations set to 2.5 million. The top 50 conformations (with the lowest binding energy) were obtained, and the conformation with the lowest binding energy was selected for further visualization. The binding parameters including binding energy, inhibition constant, intermolecular energy, total internal energy, and torsional energy were obtained from AutoDock analysis. The pdbqt file of the ligand-protein docking was input into Discovery Studio to visualize the binding residues underlying the interaction between 5-BDBD, CBD and CBV on the P2X4 receptor. The docked compounds were overlayed to visualize their arrangement in the binding pocket. Further, the binding residues were compared with the known binding residues of ATP at the P2X4 orthosteric binding site.

## 3 Results

### 3.1 Binding profile of thirteen cannabinoids with P2X4 receptor among a panel of twenty-eight compounds

**Fig. 3:**
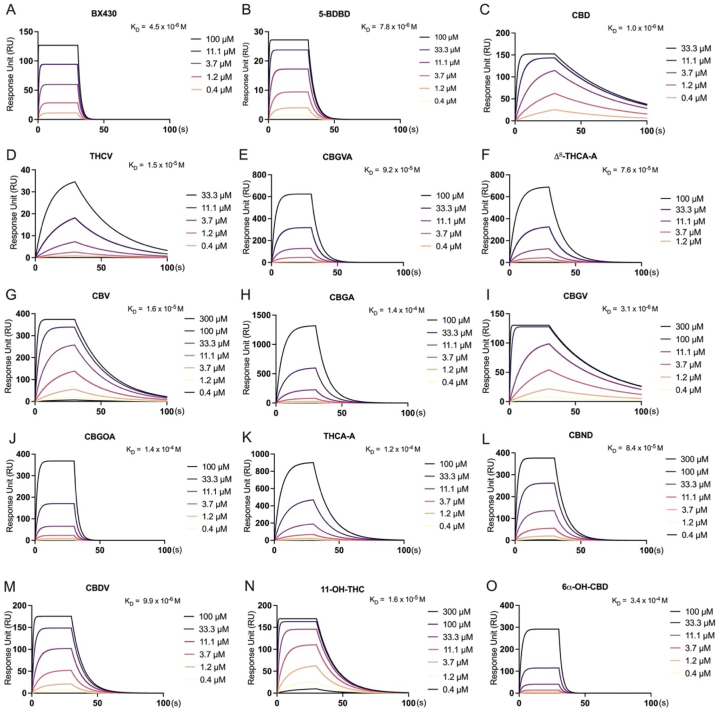
**Direct binding profiles of cannabinoids and controls with the P2X4 protein depicted by SPR sensorgrams and dissociation constants (*K***_**D**_**).** (A) BX430, (B) 5-BDBD, (C) CBD, (D) THCV, (E) CBGVA, (F) Δ^8^-THCA-A, (G) CBV, (H) CBGA, (I) CBGV, (J) CBGOA, (K) THCA-A, (L) CBND, (M) CBDV, (N) 11-OH-THC, and (O) 6α–OH–CBD.

To investigate the potential of these cannabinoids as P2X4 ligands, we conducted a screening binding assay based on SPR (Fig. 3). We successfully immobilized P2X4 protein on the CM5 chip surface, achieving a stable range of 16,000–18,000 RU for precise analyte binding affinity measurements. The theoretical Rmax values for the various cannabinoids, with such a substantial amount of ligand immobilized, were found to range from 158 to 205 RU.

The direct binding profiles of twenty-eight cannabinoids and two known P2X4 antagonists (BX430 and 5-BDBD) were assessed. To account for the unknown binding affinity of cannabinoids, a broad concentration range (0.4–300 μM) was employed. The results, illustrated in Fig. 3A and B, revealed that both BX430 and 5-BDBD exhibited moderate binding affinities for P2X4, with *K*_D_ values of 4.5 × 10^−6^ M and 7.8 × 10^−6^ M, respectively.

Among the twenty-eight cannabinoids tested, thirteen displayed weak to moderate binding affinity with P2X4, as evidenced by *K*_D_ values ranging from 3.4 × 10^−4^ M to 1 × 10^−6^ M (Fig. 3C-O). However, in the present study conducted on the Biacore T200 SPR instrument, it was observed that fifteen cannabinoids, specifically CBDAME, THCB, CBN, CBT, 11-nor-9-carboxy-THC, CBC, CBDA, CBCV, Varinolic acid, CBL, CBGM, CBG, CBDB, CBDP, and THCP, did not demonstrate any binding affinity with P2X4.

Furthermore, certain cannabinoids, such as CBGA (Fig. 3H), CBGOA (Fig. 3J), THCA-A (Fig. 3K) and 6α–OH–CBD (Fig. 3O), demonstrated binding affinity with P2X4 (with a *K*_D_ value that is higher than 10^−5^ M), while others such as CBD (Fig. 3C), CBGV (Fig. 3I), CBDV (Fig. 3M), and 11-OH-THC (Fig. 3N) displayed relatively moderate binding affinity (as suggested by a *K*_D_ value that is lower than 10^−6^ M with P2X4). Notably, during our investigation, CBD (Fig. 3C), THCV (Fig. 3D), CBV (Fig. 3G), and CBGV (Fig. 3I) exhibited a slow dissociation phase with P2X4, indicating that they were not completely removed from P2X4 during the disassociation process. Similarly, cannabinoids such as CBGVA (Fig. 3E), Δ^8^-THCA-A (Fig. 3F), CBGA (Fig. 3H), and THCA-A (Fig. 3K) demonstrated slow association and dissociation phases with P2X4. Furthermore, we observed that CBGVA (Fig. 3E), Δ^8^-THCA-A (Fig. 3F), CBGOA (Fig. 3J), THCA-A (Fig. 3K), CBND (Fig. 3L), and 6α–OH–CBD (Fig. 3O) displayed RU (Response Units) values higher than the theoretical Rmax (maximum response) values, which can be attributed to matrix binding effects. The detailed binding parameters including the binding association (K_on_) rate, the dissociation (K_off_) rate, and the binding dissociation (*K*_D_) constant are displayed in Table 1.

**Table 1:** Binding parameters of controls (BX430 and 5-BDBD) and twenty-eight cannabinoids.

Compound	K_on_ (1/Ms)	K_off_ (1/s)	*K*_D_ (M)
BX430	1.099E+5	0.4947	4.500E-6
5-BDBD	2.882E+4	0.2246	7.795E-6
CBD	1.922E+4	0.01998	1.039E-6
THCV	2175	0.03374	1.552E-5
CBGVA	1892	0.1741	9.200E-5
Δ^8^-THCA-A	1063	0.08122	7.640E-5
CBV	2474	0.04054	1.638E-5
CBGA	823.5	0.1186	1.441E-4
CBGV	8446	0.02626	3.110E-6
CBGOA	2725	0.3746	1.375E-4
THCA-A	865.5	0.1009	1.166E-4
CBND	1522	0.1282	8.420E-5
CBDV	1.209E+4	0.1198	9.908E-6
11-OH-THC	1.176E+4	0.07422	6.311E-6
6α–OH–CBD	1267	0.4273	3.374E-4
CBDAME	n.d.	n.d.	n.d.
THCB	n.d.	n.d.	n.d.
CBN	n.d.	n.d.	n.d.
CBT	n.d.	n.d.	n.d.
11-nor-9-carboxy-THC	n.d.	n.d.	n.d.
CBC	n.d.	n.d.	n.d.
CBDA	n.d.	n.d.	n.d.
CBCV	n.d.	n.d.	n.d.
Varinolic acid	n.d.	n.d.	n.d.
CBL	n.d.	n.d.	n.d.
CBGM	n.d.	n.d.	n.d.
CBG	n.d.	n.d.	n.d.
CBDB	n.d.	n.d.	n.d.
CBDP	n.d.	n.d.	n.d.
THCP	n.d.	n.d.	n.d.

Note: Binding parameters, including association rate (K_on_), dissociation rate, (K_off_), and the equilibrium dissociation constants (*K*_D_) of the controls (BX430 and 5-BDBD) and a panel of twenty-eight cannabinoids. Non-binders are included with binding parameters as not detectable (n.d.).

### 3.2 Competitive binding assay reveals the binding patterns of thirteen cannabinoids

**Fig. 4:**
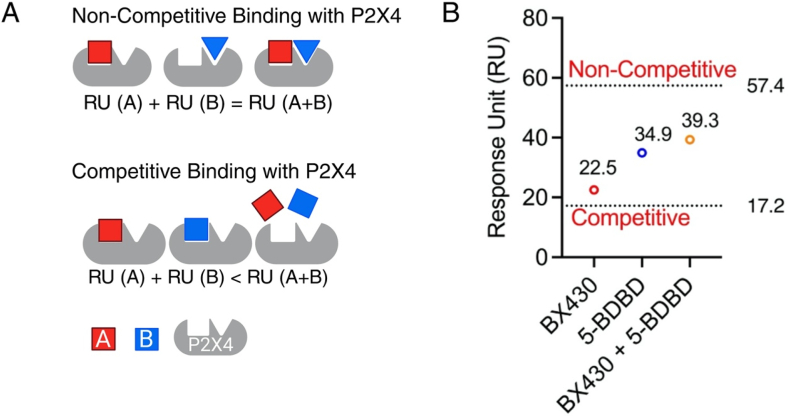
**Competitive SPR binding assay schematic.** (A) Competitive binding with P2X4 indicated by the binding mode: RU (A) + RU (B) < RU (A + B). Non-competitive binding with P2X4 indicated by the binding mode: RU (A) + RU (B)=RU (A + B). (B) Experimental binding response unit (RU) values for BX430 (22.5), 5-BDBD (34.9) and BX430 + 5-BDBD in a 1:1 ratio (39.3). The calculated theoretical RU values for non-competitive binding of A and B were 57.4, while for competitive binding of A and B, it was 17.2.

Next, we conducted a competitive binding assay using two known P2X4 antagonists, 5-BDBD and BX430, aiming to determine if 5-BDBD binds to P2X4 in a similar manner as BX430. As mentioned, if RU(A) and RU(B) are individually lower than RU (A + B), it indicates a competitive binding scenario between molecules A and B for the P2X4 receptor (Fig. 4A). Conversely, if RU(A) + RU(B) equals RU (A + B), a non-competitive binding model is suggested for A and B on the P2X4 receptor (Fig. 4A). In our assay, we observed that the experimental RU for single binders were 22.5 for BX430 and 34.9 for 5-BDBD (Fig. 4B). When these two antagonists were combined in a 1:1 ratio, the RU obtained was 39.3 (Fig. 4B). The calculated theoretical RU values were 57.4 for non-competitive binding and 17.2 for competitive binding. (Fig. 4B). Significantly, the experimental combination RU closely resembled the theoretical RU value for non-competitive binding rather than competitive binding. These results indicate non-competitive binding profiles of BX430 and 5-BDBD on the P2X4 protein. Moreover, the fact that BX430 and 5-BDBD exhibit non-competitive binding allows us to explore the potential binding pocket of cannabinoids on P2X4 effectively using these two antagonists.

**Fig. 5:**
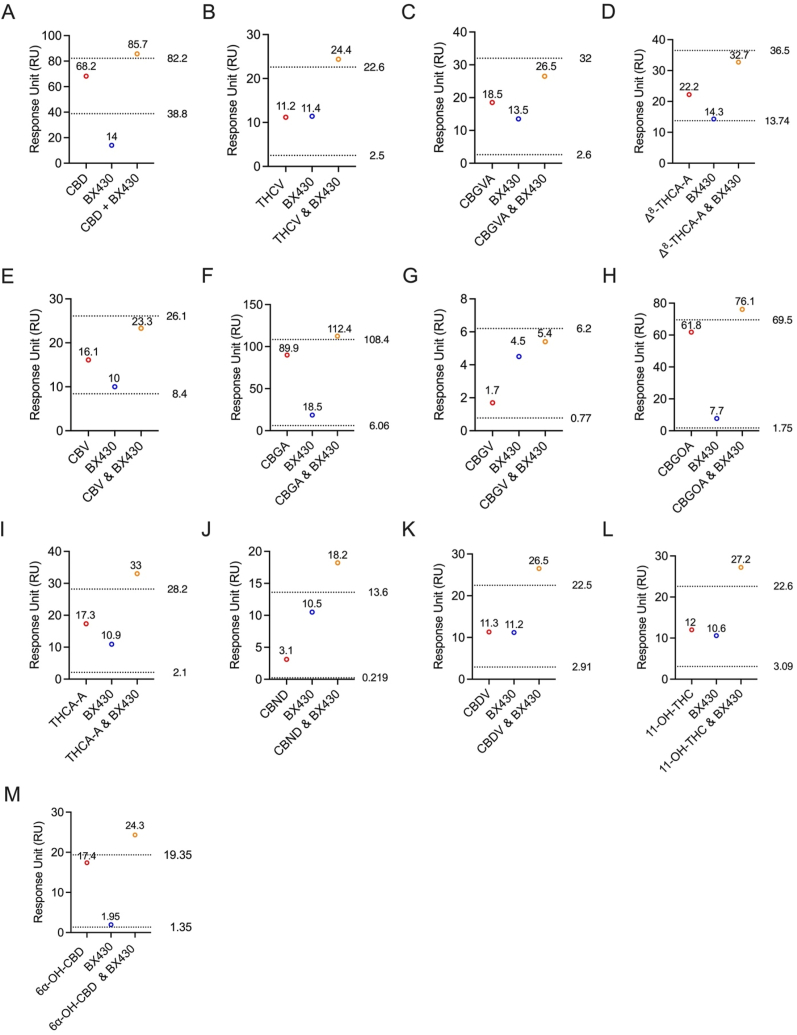
**Competitive Binding Assays of BX430 with Thirteen Cannabinoids.** Thirteen cannabinoids, namely (A) CBD, (B) THCV, (C) CBGVA, (D) Δ^8^-THCA-A, (E) CBV, (F) CBGA, (G) CBGV, (H) CBGOA, (I) THCA-A, (J) CBND, (K) CBDV, (L) 11-OH-THC, and (M) 6α–OH–CBD were individually tested along with BX430 in the competitive binding assay. Each cannabinoid was evaluated for its potential to compete with BX430 binding.

Further, we conducted two cohorts of competitive binding assays utilizing BX430 and 5-BDBD. In the initial step, we selected thirteen cannabinoids that exhibited binding affinity with P2X4 in the screening assay to be included in this competitive SPR assay. The primary objective of using BX430 in this competitive assay was to determine if the cannabinoids bind to the same allosteric site as BX430 (Fig. 5). Thirteen cannabinoids, specifically CBD (Fig. 5A), THCV (Fig. 5B), CBGVA (Fig. 5C), Δ^8^-THCA-A (Fig. 5D), CBV (Fig. 5E), CBGA (Fig. 5F), CBGV (Fig. 5G), CBGOA (Fig. 5H), THCA-A (Fig. 5I), CBND (Fig. 5J), CBDV (Fig. 5K), 11-OH-THC (Fig. 5L), and 6α–OH–CBD (Fig. 5M), identified as P2X4 binders, were individually tested.

For each corresponding sample, BX430 was independently assessed at the same concentration as the cannabinoid. The individual binding RUs were then compared to the combined 1:1 ratio of cannabinoid to BX430. The results of the competitive SPR assay, comparing the experimental and theoretical RU values, demonstrated non-competitive binding between all tested cannabinoids and BX430.

**Fig. 6:**
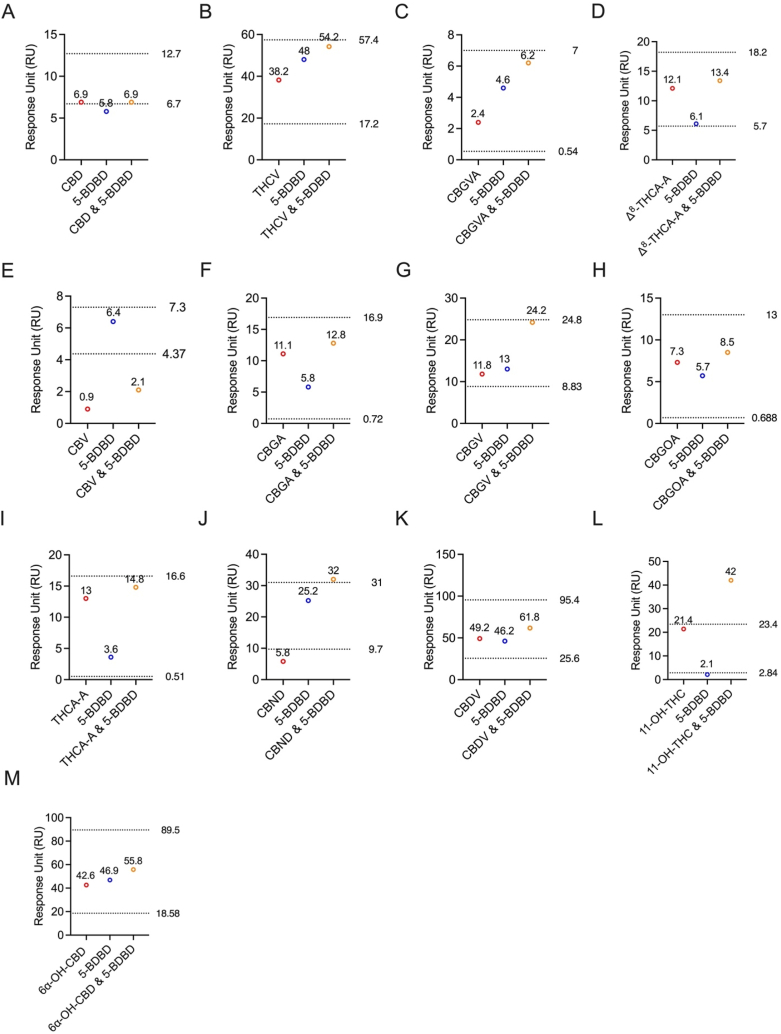
**Competitive Binding Assays of 5-BDBD with Thirteen Cannabinoids.** Thirteen cannabinoids, namely (A) CBD, (B) THCV, (C) CBGVA, (D) Δ^8^-THCA-A, (E) CBV, (F) CBGA, (G) CBGV, (H) CBGOA, (I) THCA-A, (J) CBND, (K) CBDV, (L) 11-OH-THC, and (M) 6α–OH–CBD were individually tested along with 5-BDBD in the competitive binding assay. Each cannabinoid was evaluated for its potential to compete with 5-BDBD binding.

All thirteen cannabinoids tested did not bind to the same allosteric site as BX430 on the P2X4 protein. To further investigate the binding site on the P2X4 receptor, we utilized another P2X4 antagonist, 5-BDBD, in a competitive assay. The individual binding response levels of the cannabinoids were compared with the combined 1:1 ratio of each cannabinoid with 5-BDBD (Fig. 6).

Interestingly, the results revealed that CBD (Fig. 6A) and CBV (Fig. 6E) exhibited competitive binding with 5-BDBD, as indicated by the comparison of experimental and theoretical RU values. In contrast, all other tested cannabinoids (Fig. 6B–D and Fig. 6F-M). demonstrated non-competitive binding with 5-BDBD on P2X4. Specifically, the experimental RUs for CBD and 5-BDBD were 6.9 and 5.8, respectively (Fig. 6A). When combined in a 1:1 ratio, the experimental RU was 6.9 (Fig. 6A). The theoretically calculated non-competitive RU was 12.7, while for competitive binding, it was 6.7 (Fig. 6A). Similarly, for CBV and 5-BDBD, the experimental RUs were 0.9 and 6.4, respectively (Fig. 6E). When combined in a 1:1 ratio, the experimental RU was 2.1 (Fig. 6E). The theoretically calculated non-competitive RU was 7.3, while for competitive binding, it was 4.37 (Fig. 6E). These findings provide valuable insights into the unique binding profiles of cannabinoids on the P2X4 receptor, with the majority displaying non-competitive binding characteristics with 5-BDBD.

### 3.3 Computational docking shows the binding residues of CBD and CBV on P2X4

**Fig. 7:**
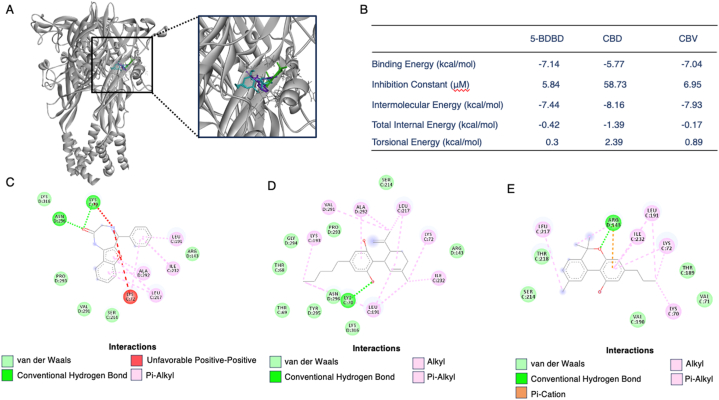
**Molecular Docking Analysis of Cannabinoids and 5-BDBD Interactions with the P2X4 Receptor.** (A) The binding interactions between cannabinoids (CBD in blue and CBV in green) and the known P2X4 antagonist, 5-BDBD (in purple), were investigated using in silico molecular docking. The individual ligand docking poses are depicted, with CBD, CBV, and 5-BDBD overlayed at the orthosteric ATP binding site of the P2X4 receptor (shown in gray). (B) Various essential binding parameters were computed to elucidate the interaction between the ligands (5-BDBD, CBD, and CBV) and the P2X4 receptor. These parameters include binding energy, inhibition constant, intermolecular energy, total internal energy, and torsional energy, providing crucial insights into the ligand-receptor interactions. (C–E) The predicted binding residues for 5-BDBD, CBD and CBV interaction with the P2X4 receptor at its orthosteric active site are shown (visualization performed using Discovery Studio).

Following the identification of the binding pocket of CBD and CBV through the competitive binding assay, we conducted molecular docking experiments to explore their interactions with the P2X4 protein. Both CBD and CBV, identified as competitive binders with 5-BDBD, were docked independently to the same pocket, exhibiting comparable binding energies of −5.77 and −7.04, respectively (Fig. 7). Visualization of all three ligands (CBD in blue, CBV in green, and 5-BDBD in purple) overlaid on the P2X4 receptor revealed a comparable binding site, indicating an overlapping arrangement of the ligands within the pocket (Fig. 7A). The inhibition constants for both ligands were also in the micromolar range, at 58.73 μM for CBD and 6.95 μM for CBV (Fig. 7B).

Next, we analyzed the binding residues for 5-BDBD, CBD, and CBV interacting with the P2X4 receptor (Fig. 7C–E). 5-BDBD binding to P2X4 involved van der Waals forces with ARG143, SER214, VAL291, PRO293, and LYS316, conventional hydrogen bonds with LYS70 and ASN296, an unfavorable bond with LYS72, and pi-alkyl bonds with LEU191, LEU17, ILE232, and ALA292 (Fig. 7C). For CBD, binding residues included van der Waals forces with THR68, THR69, ARG143, SER214, PRO293, GLY294, TYR295, ASN296, and LYS316. A conventional hydrogen bond was similarly identified at LYS70, and alkyl and pi-alkyl bonds included LYS72, LEU191, LYS193, ILE232, VAL291, and ALA292 (Fig. 7D). For CBV, the binding residues were identified as van der Waals interactions with VAL71, THR189, VAL190, SER214, and THR218. ARG143 formed a conventional hydrogen bond as well as a pi-cation interaction, and alkyl and pi-alkyl bonds included LYS70, LYS72, LEU191, LEU217, and ILE232 (Fig. 7E). These molecular docking results provided valuable insights into the interactions between CBD and CBV with the P2X4 protein, further supporting their competitive binding characteristics observed in the assay.

## 4 Discussion

There has been a limited discovery of efficacious small molecule P2X4 modulators from natural products [22]. Specifically, cannabinoids from *Cannabis* remain unexplored for the P2X4 binding capacities. Given that we reported the modulatory effect of CBD on P2X7, and further, the reported close similarity of P2X4 to P2X7, we sought to determine whether there is a direct interaction between cannabinoids and P2X4 as compared to known small molecule P2X4 antagonists, BX430 and 5-BDBD [18,24]. In this study, we established a rational workflow by employing a SPR-based platform, which included both a binding screening assay and a competitive binding assay, in combination with computational docking. The objective was to screen a panel of cannabinoids for their potential as P2X4 ligands.

In the initial phase, we established a SPR-based screening platform using a 384-well plate. This platform allowed us to efficiently screen a panel of twenty-eight cannabinoids, leading to the identification of thirteen "hit" compounds that displayed concentration-dependent response curves when binding to P2X4. Although the binding manner of 5-BDBD is contradictory in literature, data from our SPR competitive assay supported that 5-BDBD's binding site is unique from BX430 (an allosteric binder) due to their non-competitive binding profiles [21,22]. This finding offers an additional understanding the binding mechanisms of the two antagonists, namely BX430 and 5-BDBD [25]. However, due to the lack of a competitive binding assay of ATP with 5-BDBD in this study, whether the 5-BDBD binding site is competitive or non-competitive is still controversial. Consequently, the docking model is also undermined by this limitation.

In the second stage, we designed a competitive SPR assay utilizing two known P2X4 antagonists, namely BX430 and 5-BDBD. Intriguingly, our data revealed that all thirteen "hit" cannabinoids, with the exception of CBD and CBV, demonstrated non-competitive binding with both BX430 and 5-BDBD. However, CBD and CBV exhibited competitive binding with 5-BDBD. These findings offer valuable insights into the potential binding pocket of the tested cannabinoids on the P2X4 receptor and their ability to interact either in a non-competitive or competitive manner. Further, it is of interest to note that the binding site of the twenty-six non-competitive cannabinoids has not been determined, thus they may thereby act in a unique manner at an unestablished binding site on P2X4.

In the final stage, we leveraged the binding mode observed in the competitive binding assay between CBD, CBV, and 5-BDBD to further elucidate the binding pocket of CBD and CBV on the P2X4 receptor. Through in silico docking assays, we found that 5-BDBD demonstrated a strong binding energy when docked at the orthosteric site (−7.14 kcal/mol), with an inhibition constant of 5.84 μM, which closely matched the SPR binding affinity (*K*_D_ = 7.8 μM). Molecular docking also provided validation for the binding site of CBD and CBV, shedding light on the potential binding residues involved in the interaction between the ligands and P2X4. Considering the limited identified binding sites on P2X4 and the non-competitive binding indicated by competitive SPR data between BX430 and 5-BDBD, computational docking was conducted at the ATP site. Specifically, the ATP binding site was defined by the residues: LYS70, LYS72, THR189, and LYS193 (Chain B), as well as ASN296, ARG298, and LYS316 (Chain C) [23]. All three ligands (5-BDBD, CBD, and CBV) displayed binding interactions that overlapped with the known ATP binding site residues on P2X4, particularly involving LYS70 and LYS72. 5-BDBD's binding profile also included the known ATP site residues ASN296 and LYS316. For CBD, bonds formed with the residues LYS193, ASN296, and LYS316. As for CBV, its binding residues corresponded to the known ATP binding residue THR189. Consequently, molecular docking provided strong support for the binding of 5-BDBD, CBD, and CBV to the ATP site on P2X4, and further, the close binding interactions between all three ligands in the same pocket.

Our previously reported study linked CBD's anti-inflammatory effects to the modulation of P2X7, offering valuable insights into the evaluation of cannabinoids as potential P2X4 modulators [18]. However, conflicting evidence regarding interactions between P2X4 and P2X7 receptors has been reported, necessitating further investigation to determine whether cannabinoids act as dual P2X4/P2X7 binders [[26], [27], [28], [29]]. In this study, the majority of the cannabinoids with displayed P2X4 binding affinity demonstrated non-competitive binding with both antagonists, indicating potential unique allosteric binding sites. Studies on the allosteric binding sites on P2X4 are still limited [29]. However, additional allosteric binding sites have been postulated for ivermectin, which may bind at either the transmembrane domain, or in the variable region, as well as the small molecule, paroxetine, which may bind to the center of the cation channel [26]. Similar to ivermectin and paroxetine, natural products including the tested cannabinoids may bind to P2X4 at one of these hypothesized sites. Additionally, a putative binding site on P2X4 has been identified for ginsenosides, natural products from *Panax* spp. specifically located at the center vestibule region [15]. These allosteric sites provide potential locations of where the non-competitive cannabinoids may bind on P2X4. Additional studies, such as site-directed mutagenesis for example, are necessary to fully elucidate the binding site of these cannabinoids.

There is growing interest in Cannabis*-*derived compounds as potential therapeutic agents for a variety of clinical implications [30]. Currently, the number of clinical trials exploring Cannabis has seen exponential growth, due to advancements in phytochemical isolation methods and the elucidation of the endocannabinoid system [31]. While the effects of cannabinoids on cannabinoid receptor (CB)1 and CB2 has been well established, certain cannabinoids have been shown to target receptors outside of the endocannabinoid system [30,32]. Specifically, Cannabis-based medicinal properties have demonstrated promise in neurological therapeutics, such as neurodegenerative diseases, motor neuron diseases, epilepsy, autism, neuroinflammatory diseases, and neuropathic pain [16,33,34]. In this study, our findings support the modulation of receptors outside of the endocannabinoid system by major and minor cannabinoids. Binding of cannabinoids to P2X4 emphasizes the effect of Cannabis-derivatives on purinergic signaling which has been primarily characterized for other P2X receptors. Specifically, P2X4 has been shown to be expressed throughout the nervous system, modulate neurotransmitters, and is a target for multiple pathologies including inflammatory diseases, neurodegenerative diseases, and neuropathic pain [3]. Therefore, our study may provide insight into the development of cannabinoid-based therapeutics for nervous system-related conditions via mechanisms not traditionally associated with the endocannabinoid system.

This study has several limitations that should be acknowledged. Firstly, although a target-centric strategy was utilized in the SPR screening assay, the similarity in backbones among cannabinoids may have restricted the diversity of the compound library used for screening. Employing a more diverse compound library could enhance the screening assay's effectiveness. Secondly, certain cannabinoids, such as CBGVA, Δ^8^-THCA-A, CBGOA, THCA-A, CBND, and 6α–OH–CBD, exhibited matrix binding effects during the experimentation. Addressing this issue could involve employing sensor chips with different matrix compositions. For instance, exploring other types of chips, such as PEG chips, may help mitigate this challenge and yield more accurate binding units in future screenings. Thirdly, the competitive binding assay involving BX430 and 5-BDBD suggests non-competitive binding profiles on the P2X4 protein. However, it is worth noting that the RU obtained from the combination of these two antagonists was 39.3, a value closely resembling that of 5-BDBD alone (34.9 RU). Although previous references report that BX430 and 5-BDBD bind to distinct binding sites on P2X4 [8,9], further validation of our findings by additional techniques such as thermo shift experiments or nuclear magnetic resonance is warranted. Moreover, while we identified competitive binders with 5-BDBD (CBD and CBV) and non-competitive binders through this study, it is essential to conduct functional assays to characterize how these competitive and non-competitive binders modulate the biological function of the P2X4 receptor. Such assays are crucial in determining whether the observed binding behaviors have an impact on P2X4 signaling. Therefore, further investigations utilizing cell-based and animal models are warranted to validate the finding that P2X4 is a target of CBD and other minor cannabinoids. These follow-up studies are critical for advancing the development of cannabinoids as potential therapeutics.

## 5 Conclusion

In conclusion, our study identified thirteen cannabinoids as P2X4 binders using a novel SPR-based screening platform. We characterized the binding profiles of these binders by utilizing the known P2X4 antagonists BX430 and 5-BDBD as probes. Notably, CBD and CBV were identified as competitive binders with 5-BDBD on P2X4, while all other cannabinoids displayed non-competitive binding. Molecular docking experiments further supported the overlapping interactions of 5-BDBD, CBD, and CBV in the same binding pocket. This study provides the first insight into the binding behavior of cannabinoids to P2X4, highlighting their potential as P2X4 modulators which may have implications for Cannabis derivative therapeutic development against neuroimmune, neurodegenerative, and/or pain conditions.

## Data availability statement

Data in this study have not been deposited into a publicly available depository. Data will be made available upon reasonable request.

## CRediT authorship contribution statement

**Tess Puopolo:** Writing – original draft, Investigation, Formal analysis. **Ang Cai:** Writing – review & editing, Formal analysis. **Chang Liu:** Writing – review & editing, Writing – original draft, Methodology, Investigation, Formal analysis, Data curation, Conceptualization. **Hang Ma:** Writing – review & editing, Conceptualization. **Navindra P. Seeram:** Writing – review & editing, Supervision, Resources, Funding acquisition.

## Declaration of competing interest

The authors declare that they have no known competing financial interests or personal relationships that could have appeared to influence the work reported in this paper.
